# Supplementary material for: Identification of photoperiod-induced specific miRNAs in the adrenal glands of Sunite sheep (Ovis aries)
Source: Front Vet Sci. 2022 Jul 22;9:888207. doi: 10.3389/fvets.2022.888207 (PMC9354845; doi:10.3389/fvets.2022.888207)
Supplement: Supplementary file 1 [file Data_Sheet_1.ZIP › Supplementary file/miRDeep2 file.zip!/miRDeep2 file/Know_Structure/SPLP42A1/oar-mir-543.pdf]

miRBase precursor : oar-mir-543
Total read count : 15475
oar-miR-543-5p read count : 10
oar-miR-543-3p read count : 15435
remaining reads : 30

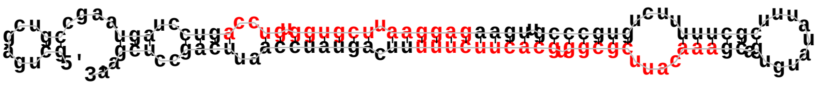

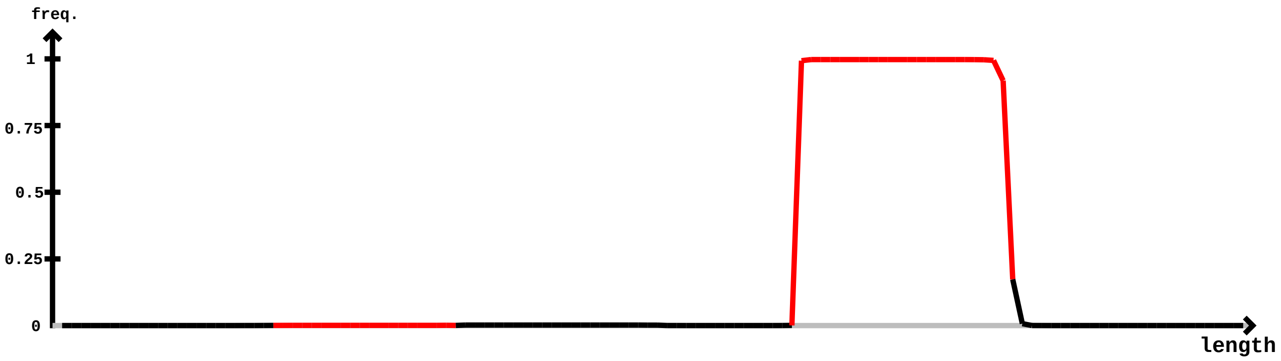


```
                                                                              oar-miR-543-3p
                     oar-miR-543-5p
5'- gcugagcugccgaaugauccugaccuguggugcuuaaggagaaguugcccgugucuuuuucgcuuuauaugugacgaaacauucgcggugcacuucuuuuucaguauccaauucagccucgaa  -3'   exp
    ((......)).....((..((((..((.((((((.((((((((((.(((((((....((((((.........).)))))....))))).)))))))))))).))))))))..))))..))...   reads   mm
    ..................ccugaccuguggugcuuaagga...................................................................................   1       0
    ....................ugaccuguggugcuuaagga...................................................................................   4       0
    .....................gaccuguggugcuuaagga...................................................................................   1       0
    .....................gaccuguggugcuuaaggag..................................................................................   1       0
    ......................accuguggugcuuaagga...................................................................................   1       0
    ......................accuguggugcuuaaggag..................................................................................   1       0
    .......................ccuguggugcuuaagga...................................................................................   2       0
    .......................................agaaguugcccgugucuuuuu...............................................................   3       0
    .......................................agaaguugcccgugucuuuuucg.............................................................   1       0
    ........................................gaaguugcccgugucuuuuucg.............................................................   1       0
    .........................................aaguugcccgugucuuuuu...............................................................   1       0
    .........................................aaguugcccgugucuuuuuc..............................................................   1       0
    .........................................aaguugcccgugucuuuuucg.............................................................   21      0
    .........................................aaguugcccgugucuuuuucgcu...........................................................   1       0
    ..........................................................................cgaaacauucgcggugcacuucu..........................   2       0
    ...........................................................................gaaacauucgcggugcacuucu..........................   7       0
    ...........................................................................gaaacauucgcggugcacuucuu.........................   1       0
    ............................................................................aaacauucgcggugcacu.............................   4       0
    ............................................................................aaacauucgcggugcacuu............................   7       0
    ............................................................................aaacauucgcggugcacuuc...........................   37      0
    ............................................................................aaacauucgcggugcacuucu..........................   1161    0
    ............................................................................aaacauucgcggugcacuucuu.........................   11490   0
    ............................................................................aaacauucgcggugcacuucuuu........................   2552    0
    ............................................................................aaacauucgcggugcacuucuuuu.......................   106     0
    ............................................................................aaacauucgcggugcacuucuuuuu......................   2       0
    ............................................................................aaacauucgcggugcacuucuuuuuc.....................   1       0
    .............................................................................aacauucgcggugcacuucu..........................   3       0
    .............................................................................aacauucgcggugcacuucuu.........................   42      0
    .............................................................................aacauucgcggugcacuucuuu........................   18      0
    .............................................................................aacauucgcggugcacuucuuuu.......................   2       0
```
